# Fluid inclusion petrology and microthermometry of the Cocos Ridge hydrothermal system, IODP Expedition 344 (CRISP 2), Site U1414

**DOI:** 10.1002/2015GC006212

**Published:** 2016-04-26

**Authors:** Jennifer Brandstätter, Walter Kurz, Kurt Krenn, Peter Micheuz

**Affiliations:** ^1^ University of Graz, Institute of Earth Sciences, NAWI Graz GeocenterGrazAustria

**Keywords:** fluid inclusions, hydrothermal veins, erosive plate boundary, Cocos Ridge, IODP Expedition 344 Site U1414, hotspot

## Abstract

In this study, we present new data from microthermometry of fluid inclusions entrapped in hydrothermal veins along the Cocos Ridge from the IODP Expedition 344 Site U1414. The results of our study concern a primary task of IODP Expedition 344 to evaluate fluid/rock interaction linked with the tectonic evolution of the incoming Cocos Plate from the Early Miocene up to recent times. Aqueous, low saline fluids are concentrated within veins from both the Cocos Ridge basalt and the overlying lithified sediments of Unit III. Mineralization and crosscutting relationships give constraints for different vein generations. Isochores from primary, reequilibrated, and secondary fluid inclusions crossed with litho/hydrostatic pressures indicate an anticlockwise *PT* evolution during vein precipitation and modification by isobaric heating and subsequent cooling at pressures between ∼210 and 350 bar. Internal over and underpressures in the inclusions enabled decrepitation and reequilibration of early inclusions but also modification of vein generations in the Cocos Ridge basalt and in the lithified sediments. We propose that lithification of the sediments was accompanied with a first stage of vein development (VU1 and VC1) that resulted from Galapagos hotspot activity in the Middle Miocene. Heat advection, either related to the Cocos‐Nazca spreading center or to hotspot activity closer to the Middle America Trench, led to subsequent vein modification (VC2, VU2/3) related to isobaric heating. The latest mineralization (VC3, VU3) within aragonite and calcite veins and some vesicles of the Cocos Ridge basalt occurred during crustal cooling up to recent times. Fluid inclusion analyses and published isotope data show evidence for communication with deeper sourced, high‐temperature hydrothermal fluids within the Cocos Plate. The fluid source of the hydrothermal veins reflects aqueous low saline pore water mixed with invaded seawater.

## Key Points

Different fluid inclusion generations give insights into the evolution of IODP Site 344‐U1414
Advective heat transport, probably related to the Galapagos hotspot or a spreading center close to Cocos Island, effectuated lithification of sediments of the Cocos Plate

## 1 Introduction

The Integrated Ocean Drilling Program (IODP) Expedition 344 as part of the Costa Rica Seismogenesis Project (CRISP) is focused on lithology observation, fluid characterization, and temperature and stress indication at the transition zone from stable to unstable slip along the Cocos Plate—Caribbean Plate boundary [*Harris et al*., 2013a]. Fundamental to this objective is an understanding of the nature of the sediments and oceanic crust entering the seismogenic zone, the hydrologic system, and the thermal state of the subducting igneous oceanic crust. For this approach, Site 344‐U1414 (proposed Site CRIS‐19A) serves as a reference site on the flank of the subducting aseismic Cocos Ridge.

The oceanic Cocos Plate contributes to the evolution of the hydrologic system by means of fluids which trapped and circulated along fractures as well as chemically bound fluids preserved in the alteration products. Pathways of fluid flow at the Costa Rica margin therefore also include the incoming oceanic crust. Characterizing the fluid at the source and monitoring fluid pressure, chemistry, and temperature, will help us to understand the temporal relationships among stress, strain, and pore fluid composition at the plate boundary [e.g., *Silver et al*., 2000; *Fisher et al*., 2003; *Sahling et al*., 2008; *Harris et al*., 2010a, 2010b].

In this study, we provide data from microthermometry of fluid inclusions entrapped in hydrothermal veins within the Cocos Ridge basalt and the lithified sediments of Unit III from IODP Expedition 344 Site U1414 and examine a primary task of Expedition 344, i.e., to evaluate fluid/rock interaction, the hydrologic system, and the geochemical processes (controlled by composition and volume of fluids) active within the lower Cocos Plate. The origin of high temperature fluids and possible heat sources will be discussed.

## 2 Geological Background and Plate Tectonic Setting

The Central America margin offshore Costa Rica is one of the best studied subduction zones with a wide spectrum of data from seismicity, onland geology, volcanic petrology, geodesy, seismic imaging, submersible dives, and four deep‐sea drilling and long‐term monitoring cruises (DSDP Leg 84, ODP Leg 170 and 205, IODP Leg 301T). The CRISP drilling area is located in a region where the incoming plate has a relatively thin sedimentary cover, large variations in bathymetry along‐strike, and a fast convergence rate. This active continental margin is a sediment‐poor subduction zone with a history of Mw > 7 earthquakes and active tectonic erosion [e.g., *Harris et al*., 2012].

**Figure 1:**
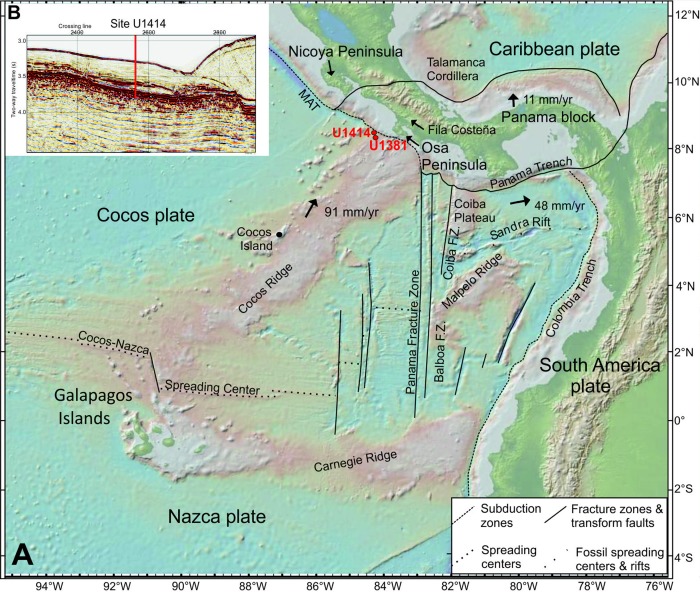
(a) Digital elevation map of the Costa Rica area [http://www.geomapapp.org ] [*Ryan et al*., 2009], showing the locations of drilling Site U1414 and Site U1381of IODP Expedition 344 and the context to the general plate tectonic setting. F.Z. – fracture zone; MAT‐ Middle America Trench. (b): Seismic travel time section of Line 2497 with location of Site U1414 [after *Harris et al*., 2013b].

Offshore the western margin of Costa Rica, the oceanic Cocos Plate subducts under the Caribbean Plate at the southern end of the Middle America Trench (Figure 1). All subduction parameters including the age, convergence rate, azimuth, obliquity, morphology, and slab dip vary along strike. The age of the Cocos Plate at the Middle America Trench decreases from 24 Ma offshore Nicoya Peninsula to 15 Ma offshore Osa Peninsula [*Barckhausen et al*., 2001] (Figure 1). Subduction rates vary from 70 mm/y offshore Guatemala to 90 mm/y offshore southern Costa Rica. Convergence obliquity across the trench varies from offshore Nicaragua, where it is as much as 25° oblique to nearly orthogonal southeast of the Nicoya Peninsula [*Turner et al*., 2007]. The bathymetry and morphology of the incoming Cocos Plate are largely a function of its origin and subsequent history. The Cocos Plate was formed at two ridges, the fast‐spreading East Pacific Rise (EPR) and the slow‐spreading Cocos‐Nazca spreading center (CNS). The boundary separating EPR from CNS crust is a combination of a triple junction trace and a fracture zone, collectively comprising a “plate suture.” EPR‐generated crust has a generally smoother morphology than CNS‐generated crust. Cocos Plate lithosphere offshore Costa Rica formed at the CNS‐1 (∼23 Ma) and records two southward ridge jumps at 19.5 (CNS‐2) and 14.5 Ma (CNS‐3) [*Barckhausen et al*., 2001]. Passage of the Cocos Plate over the Galapagos hotspot (Figure 1) created the aseismic Cocos Ridge (CCR). The 13‐14.5 Ma CCR [*Werner et al*., 1999; *Harpp et al*., 2005] and seamounts are significant morphological segments of the Cocos Plate. The CCR is ∼25 km thick, stands 2.5 km high and is characterized by a distinctive Galapagos‐type geochemistry [e.g., *Hoernle et al*., 2002; *Vannucchi et al*., 2013; *Schindlbeck et al*., 2015]. Northwest of the CCR, ∼40% of CNS generated oceanic crust is covered by seamounts that also have a Galapagos‐type geochemistry. These seamounts increase the roughness of the seafloor and have likely caused substantial subduction erosion of the outer forearc [*Ranero and von Huene*, 2000] as well as uplift of the Osa and Nicoya Peninsulas [*Gardner et al*., 1992, 2001; *Fisher et al*., 1998; *Sak et al*., 2004]. In the area of Osa Peninsula, the overthickened CCR is more buoyant than normal oceanic crust and causes a shallowing of the Wadati‐Benioff Zone. The seismically active slab dips ∼65° near the Nicaraguan border and shallows to a few degrees inboard of the CCR [*Vergara Muñoz*, 1988; *Protti et al*., 1994].

## 3 U1414 Site Location: Stratigraphy

**Figure 2:**
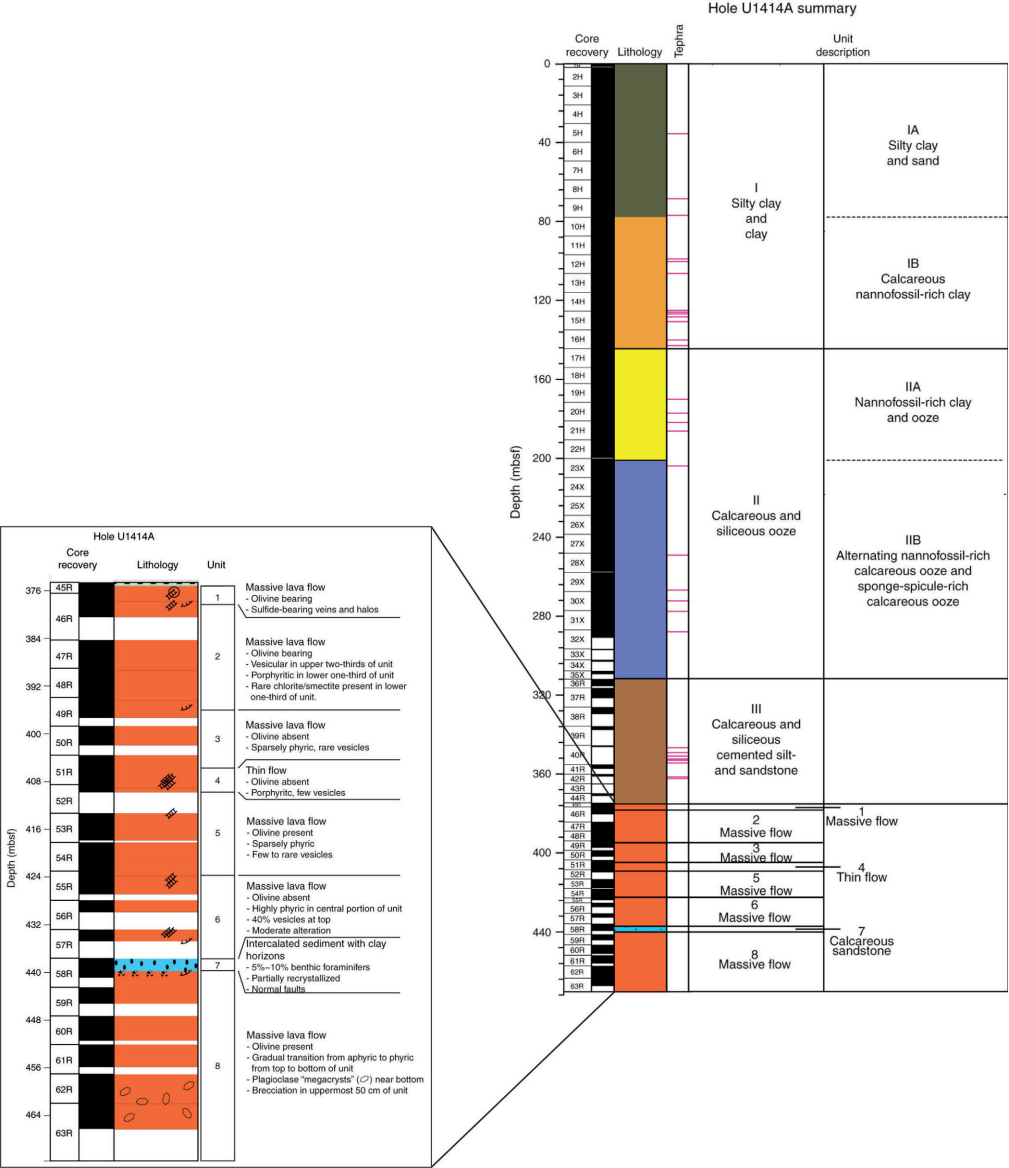
Stratigraphy of Site 344‐U1414. The cored interval was divided into three major sedimentary units with four subunits and eight igneous basement units with one intercalated sedimentary layer [after *Harris et al*., 2013b].

Site 344‐U1414 is located ∼1 km seaward of the Middle America Trench (Figure 1) and was drilled to investigate the lithostratigraphy and pore water of the sedimentary sequence and the uppermost portions of the underlying CCR basalt. In Hole U1414A, cored interval was divided into three sedimentary units with four subunits and eight basement units with one thin intercalated sedimentary layer (Figure 2). These units comprise 375.25 m of sediment above 96.35 m of igneous basement. Overall core recovery was 86%: 96% for APC coring, 86% for XPC coring, and 34% for RCB coring [see *Harris et al*., 2013a, for details].

The top of Hole U1414A is characterized by a predominantly monotonous sequence of soft, light greenish gray hemipelagic silty clay to clay. The uppermost part of Unit I (Subunit IA) contains several thin sand layers, whereas calcareous nannofossils gradually increase in the lower part (Subunit IB). Unit I contains terrigenous material (lithic fragments, glass shards, and minerals) that decreases with depth. Biogenic material such as foraminifers and radiolarians is abundant. Tephra layers make up ∼1% of Unit I and are distributed into 16 well‐sorted, discrete tephra horizons. The Unit I—Unit II boundary at 145.34 m below seafloor (mbsf) (section 344‐U1414A‐17H‐1, 94 cm) is marked by a change from greenish gray nannofossil‐rich clay sediment to brownish to whitish nannofossil‐rich calcareous ooze. Unit II (145.34 mbsf; sections 344‐U1414A‐17H‐1, 94 cm, to 35X‐CC, 29 cm) is a 164.03 m thick, moderately consolidated, white to dark grayish to yellowish brown clayey to silty interval that is divided into two subunits. Unit II is generally composed of nannofossil‐rich calcareous ooze and variable amounts of sponge spicules, foraminifers, and diatoms. Subunit IIA (145.34–200.01 mbsf; sections 344‐U1414A‐ 17H‐1, 94 cm, to 22H‐CC, 30 cm) is dominated by calcareous nannofossil ooze, whereas Subunit IIB is characterized by meter‐scale alternating calcareous nannofossil ooze and biosilica‐rich calcareous ooze.

**Figure 3:**
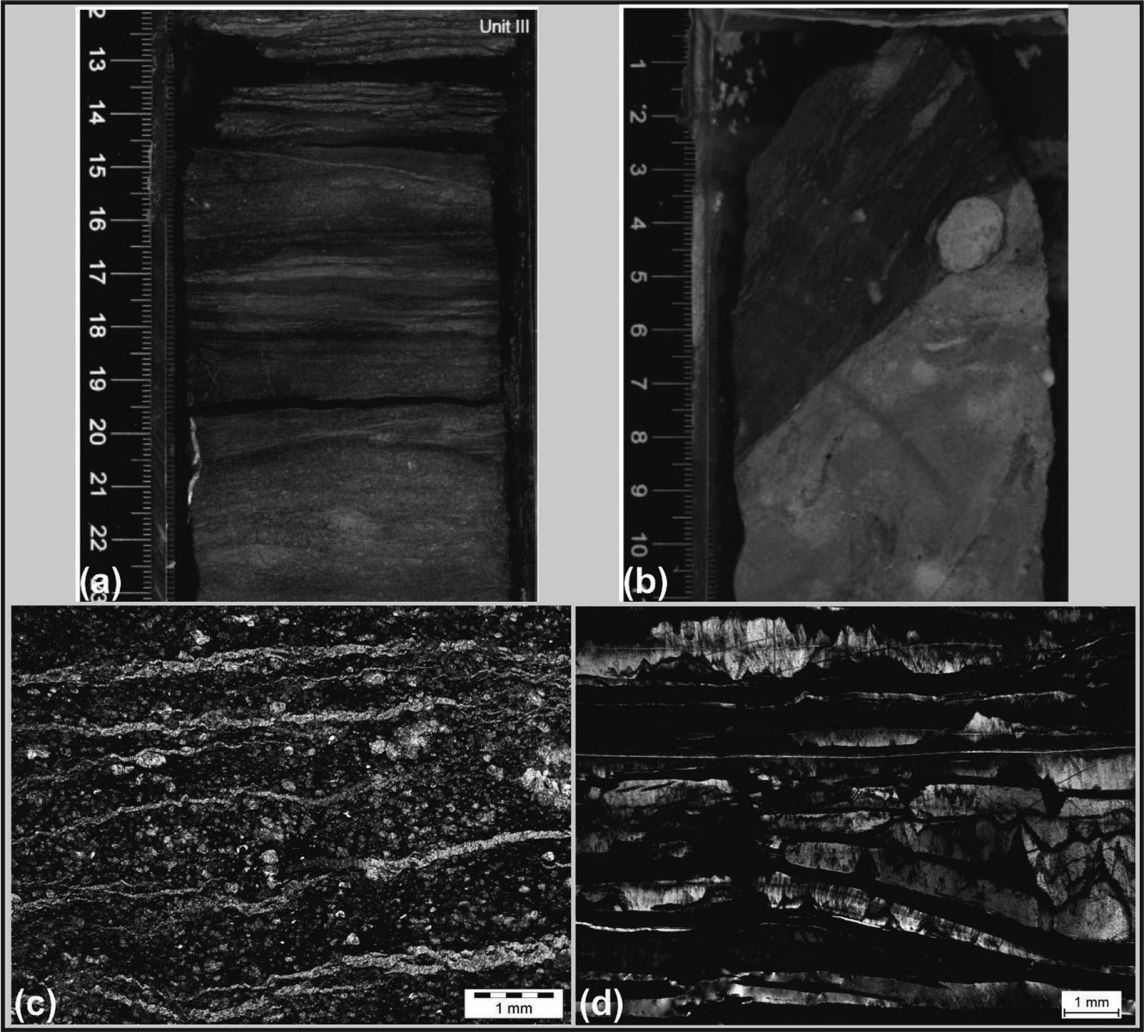
(a) Representative digital image of lithostratigraphic Unit III, showing brownish to very dark gray siliceous sandstone interlayered by chert‐like laminae (bright gray layers). Sedimentary structures, probably representing former primary bedding, have been replaced by diagenetic silica (interval 344‐U1414A‐41R‐2A, 12–23 cm). (b) Sharp inclined contact of calcareous to siliceous siltstone in Unit III to completely lithified limestone breccia consisting of centimeter‐sized clasts (interval 344‐U1414A‐42R‐CCA, 0–11 cm). (c‐d) Microstructures of veins in the sedimentary rocks of Unit III (crossed polars): (c) Bedding planes filled by calcite (sample JB35). (d) Calcite veins with distinct stylolites (sample JB40).

Unit III is a 65.88 m thick interval between 309.37 and 375.25 mbsf (sections 344‐U1414A‐35X‐CC, 29 cm, to 45R‐1, 65 cm). It is a sequence of lithified, calcareous, and siliceous cemented silt and sandstone with well‐preserved original sedimentary structures such as bedding and bioturbation. The boundary between Units II and III is marked by a noticeable increase in the lithification state of the sediments, also obvious from different physical properties data (e.g., strength, thermal conductivity), a change to a dark reddish brown color, and the occurrence deformational structures like foliation (Figure 3a), faults and mineralized veins [*Harris et al*., 2013a]. The matrix of the strongly lithified calcareous and siliceous silt to sandstone is replaced by cement. The spacings between bedding and foliation planes are also filled by calcite and silica (Figure 3a). Formation of a foliation is also evident from flow structures of fine‐grained matrix material around (siliceous) rounded clasts (Figure 3b). The main mineralogic components appear to be the recrystallized sedimentary components of former silt and sandstones. In section 344‐U1414A‐42RCC, a sharp inclined contact to completely lithified limestone breccia made out of centimeter‐sized clasts is nicely preserved (Figure 3b). The biogenic components in this unit have been lost, most likely because of lithification and recrystallization. Tephra layers and pods comprise <1% of this interval. The lower part of Unit III is characterized by well‐developed foliation with dip angles ranging from subhorizontal to moderately inclined. The foliation is characterized by millimeter‐scale spacing and predominantly outlined by calcite layers and distinct stylolites (Figures 3c and 3d). In distinct domains, coarse (siliceous) clasts derived from bioturbation structures are embedded within the fine‐grained foliated matrix. Around these clasts, flow structures, similar to pressure shadows, can be observed. The foliation is crosscut by calcite‐filled veins with various orientations. The wall rocks along the veins are often fractured into fragments or are even brecciated. Additionally, high‐angle normal faults are associated with the veins. No veins were observed within Units I and II.

A sedimentary interval interbedded within the basalt between 437 and 439.37 mbsf is characterized by horizontal to subhorizontal sedimentary structures (lamination and foliation) and a small population of mineral veins. The sediment is mainly composed of asymmetrical sandstone lenses embedded in a finer grained matrix.

The igneous CCR basement was drilled from 375.25 to 471.6 mbsf (0–96.4 m subbasement (msb)) (Figure 2), of which 61.6 m was recovered (66%). It broadly comprises aphyric to highly phyric massive basaltic flows and thin flows that are divided into seven units, with one additional unit of intercalated calcareous sandstone (see paragraph above). Structures in the tholeiitic CCR basalt (CCR) mainly comprise mineralized veins at various orientations. A preferred orientation of strike directions was not observed.

## 4 Methods

Fluid inclusions (FIs) were investigated on doubly polished rock sections from samples of IODP Site 344‐U1414 (Table 1) with a thickness of ∼ 0.20–0.30 mm using a LINKAM THSMG600 heating and freezing stage with an operating range from −196°C to +600°C, at the Institute of Earth Sciences, University of Graz. The Synthetic Fluid Inclusion Reference Set (Bubbles Inc. Blackburg, VA, USA 24062‐0146) was used for stage calibration. Temperature measurements are reproducible within 0.2°C at a heating rate of 0.1°C/min. Fluid salinities and densities were calculated with the software BULK by using the appropriate equations of state after *Bodnar* [1993] for aqueous inclusions. Isochores were calculated with the software ISOC using the equations of state after *Bodnar and Vityk* [1994]. The programs BULK and ISOC are included in the software package FLUIDS 1 [*Bakker*, 2003]. All FIs were initially cooled to below −100°C and subsequently heated to determine the temperatures of phase transitions. Depending on the compositional system for any given FI, the following values are documented (abbreviations after *Diamond*, 2003]: *T*_e_(Ice) temperature of eutectic melting of ice (IceV → IceLV); *T*_e_(Ice) was used to identify the saline aqueous fluid system after *Davis* [1990] and *Goldstein and Reynolds* [1994]; *T*_m_(Ice) temperatures of final melting of ice (IceLV → LV); *T*_m_(Ice) was observed to calculate salinities of aqueous fluid inclusions using freezing point depression as well as equations according to *Bodnar* [1993]. Total homogenization temperature *T*_h_(total) (LV → L or V) was measured to get minimum conditions for formation of homogenously trapped FIs. All analyzed FIs homogenize into the liquid phase. Due to the low eutectic temperatures *T*_e_(Ice) down to −70°C in all studied FIs in the veins, H_2_O‐NaCl fluid chemistry with additional chlorides like KCl, MgCl_2_, and CaCl_2_ is suggested (Table 2). Due to the small size of FIs, cleavage, birefringence, and calcite twinning, which hampered additional phase identifications like e.g., hydrohalite, calculations were performed in the reduced system H_2_O‐NaCl. All microthermometry data are given in Table 2. Terminology of fluid inclusions arranged along planes (fluid inclusion planes: FIPs) is after *Vollbrecht et al*. [1991] modified by *Van den Kerkhof and Hein* [2001]. Abbreviations: L = liquid, V = vapor.

**Table 1:** Sample Codes; the Related IODP Sample Requests are 1607IODP and 1911IODP

Sample No.	Sample Code IODP Expedition 344	Lithology/Unit	Vein Material
JB35	344‐U1414A‐40R‐1‐W 62/65‐	Unit III	Calcite
JB36	344‐U1414A‐40R‐CC‐W 5/7‐	Unit III	Quartz
JB37	344‐U1414A‐41R‐1‐W 8/10‐	Unit III	Calcite
JB40	344‐U1414A‐41R‐2‐W 12/13‐	Unit III	Calcite
JB41	344‐U1414A‐41R‐2‐W 57/63‐	Unit III	Calcite
JB43	344‐U1414A‐41R‐2‐W 73/77‐	Unit III	Calcite, quartz
JB50	344‐U1414A‐42R‐1‐W 13/15	Unit III	Calcite
JB51	344‐U1414A‐42R‐1‐W 21/24‐	Unit III	Calcite
JB57	344‐U1414A‐44R‐1‐W 12/14‐	Unit III	Calcite
JB58	344‐U1414A‐44R‐1‐W 21/24‐	Unit III	Calcite
JB59	344‐U1414A‐45R‐2‐W 3/5‐	CCR basalt	Aragonite
JB60	344‐U1414A‐45R‐2‐W 56/64‐	CCR basalt	Quartz
JB62	344‐U1414A‐46R‐2‐W 32/34‐	CCR basalt	Aragonite
JB63	344‐U1414A‐46R‐3‐W 86/90‐	CCR basalt	Aragonite
JB66	344‐U1414A‐48R‐1‐W 88/92‐	CCR basalt	Aragonite
JB69	344‐U1414A‐49R‐1‐W 105/111‐	CCR basalt	Aragonite
JB71	344‐U1414A‐51R‐1‐W 14/18‐	CCR basalt	Quartz, calcite
JB74	344‐U1414A‐53R‐1‐W 126/131‐	CCR basalt	Quartz, calcite
JB78	344‐U1414A‐57R‐1‐W 38/43‐	CCR basalt	Quartz, calcite
JB79	344‐U1414A‐57R‐1‐W 47/53‐	CCR basalt	Quartz
JB81	344‐U1414A‐57R‐1‐W 71/76‐	CCR basalt	Calcite
JB84	344‐U1414A‐57R‐2‐W 46/52‐	CCR basalt	Calcite
JB88	344‐U1414A‐61R‐1‐W 45/49‐	CCR basalt	Calcite
JB89	344‐U1414A‐61R‐1‐W 62/69‐	CCR basalt	Aragonite
JB94	344‐U1414A‐62R‐2‐W 140/141‐	CCR basalt	Quartz

**Table 2:** Microthermometric Data of Different Fluid Inclusion Types of Unit III and the CCR Basalt^a^

Sample	Vein Type	Host	n	FI	Textural Appearance	Chemistry	Size (µm)	Phases	*T*_e_ (Ice) (°C)	*T*_m_ (ice) (°C)	*T*_h_ (Total) (°C)	Density (g/cm^3^)	Salinity (Mass%)
CCR basalt													
* JB60*	VC2	Quartz	6	1A	Single; isolated; decrep.		10–80	Laq + V	−62.0	−2.0	270–400	0.79–0.49	3.4
				2A	Cluster	H_2_O‐NaCl ± CaCl_2_ ± KCl ± MgCl_2_	<5–10	Laq + V	n.o.	n.o.	n.o.		
			7	2B	Cluster		<5–15	Laq + V	n.o.	−1.5	120–250	0.96–0.82	2.6
* JB94*	Vesicle	Quartz	6	2B	Cluster	H_2_O‐NaCl ± CaCl_2_ ± KCl ±MgCl_2_	<5–15	Laq + V	−54.0	−3.0	40–123	1.0–0.97	5.0
* JB62*	VC3	Aragonite	43	2B	Cluster;	H_2_O‐NaCl ± CaCl_2_ ± KCl ± MgCl_2_	<5–10	Laq + V	−45.0 to−‐41.0	−2.0 to −1.7	80–200	0.98–0.89	2.9–3.4
* JB66*	VC3	Aragonite	13	1B	Intragranular FIPs; elongated	H_2_O‐NaCl ± CaCl_2_ ± KCl ± MgCl_2_	6–25	Laq + V	−50.0	−1.6	260–375	0.80–0.55	2.7
			16	2B	Cluster		<5–10	Laq + V	−50.0	−1.6 to −0.5	86–215	0.98–0.83	0.8‐2.7
* JB69*	VC3	Aragonite	8	1B	Planes; intragranular FIPs;	H_2_O‐NaCl ± CaCl_2_ ± KCl ± MgCl_2_	10–20	Laq + V	−42.0	−0.2 to 0.0	260–280	0.78–0.74	0.0–0.3
			3	2B	Cluster		<5–10	Laq + V	n.o.	0.0	118–218	0.94–0.85	0.0
Unit III													
*JB37*	VU1	Calcite	7	1A	Single; decrep.		6–20	Laq + V	−45.0	−2.0	300–360	0.74–0.60	3.4
			6	2A	Cluster	H_2_O‐NaCl ± CaCl_2_ ± KCl ± MgCl_2_	<5–20	Laq + V	n.o.	n.o.	230–274		
			22	2B	Intragranular FIPs		<5–20	Laq + V	−52.0 to −45.0	−0.5	80–200	0.98–0.89	0.9
*JB50*	VU2	Calcite	16	1A	Single; decrep.		20–80	Laq + V	−52.0 to −48.0	−1.5 to −0.5	230–430	0.83–0.36	0.8–2.6
			47	2A	Satellites	H_2_O‐NaCl ± CaCl_2_ ± KCl ± MgCl_2_	<5–50	Laq + V	−54.0 to −51.0	−1.3 to −0.5	230–370	0.83–0.55	0.8–2.2
			51	2B	Intragranular FIPs		<5–30	Laq + V	−55.0 to −51.0	−3.0 to −0.3	94–200	0.97–0.89	0.5–5.0
*JB51*	VU3	Calcite	6	1A	Decrep. cluster	H_2_O‐NaCl± CaCl_2_ ± KCl ± MgCl_2_	10–60	Laq + V	−70.0 to −46.0	−2.0 to −1.5	230–373	0.83–0.57	2.6–3.4
			12	2B	Cluster		5–10	Laq + V	−40.0	−1.2 to −1.3	120–200	0.96–0.89	2.2–2.1
*JB58*	VU2	Calcite	23	2B	Cluster, FIPs elongated	H_2_O‐NaCl ± CaCl_2_ ± KCl ± MgCl_2_	<5–15	Laq + V	−55.0 to −45.0	−2.8 to −1.9	74–171	0.99–0.93	3.2–4.6

a Abbreviations: n = number of measured FIs; *T*_e_(ice) = temperature of eutectic melting of ice; *T*_m_(ice) = temperature of final melting of ice; *T*_h_(total) = total homogenization temperature; n.o. = not observed; FI types are explained in the text.

## 5 Sample Description

FI data derived from (1) quartz and calcium carbonate‐filled veins and vesicles in the CCR basalt and (2) from calcite‐filled veins in the lithified sedimentary rocks of Unit III of Hole 344‐U1414A (sampling depth ranges from 350 to 471 mbsf). The related IODP sample requests are 1607IODP and 1911IODP (Table 1).

The vein widths vary from 0.1 to 1.5 cm. Veins are composed of clay minerals like saponite, smectite, quartz, carbonate (aragonite, calcite), and pyrite. Some veins show straight boundaries, others are characterized by irregular wall geometries.

**Figure 4:**
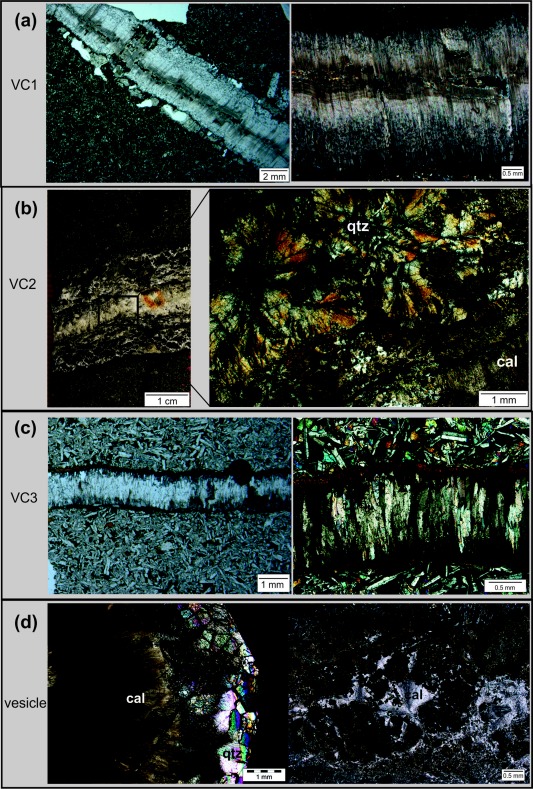
Structures of veins within the Cocos Ridge basalt: (a) Blocky antitaxial vein (VC1) with fibrous and acicular calcite crystals. Note the characteristic median line consisting of wall rock fragments, clay minerals, and quartz (sample JB 84). (b) Hand specimen of polymineralic vein type VC2 (sample JB78). (left) Quartz forms the center of the vein; the outer area is composed of clay minerals and fibrous calcite. (right) Microstructure of sample JB78 (crossed polars): the center is filled by spheroidal radiating quartz grains, mostly overgrown by calcite, and surrounded by clay minerals (brown) and calcite. (c) Syntaxial vein (VC3) filled by elongated aragonite with a clay selvage (brown) (sample JB59). (left: d) Microstructure of vesicle filled with quartz and overgrown by acicular calcite (sample JB94, crossed polars). (right) Vesicle filled with clay minerals and fibrous calcite (sample JB65, crossed polars). See Table 1 for sampling codes.

In the CCR basalt veins consistently have various dip angles, at greater depths the veins become steeper. The veins rarely show monomineralic filling. According to the microstructural observations and occurrence of the vein‐filling minerals, the following sequence of vein generations, from oldest to youngest, is defined for the CCR basalt: (1) clay minerals + calcite, (2) quartz, (3) calcium carbonate. Crystal growth within all veins is generally perpendicular to vein boundaries and indicate the direction of extension, except spherulitic quartz and calcite crystals. Following the classification of *Bons et al*. [2012], three different types of veins can be distinguished. *Type one (VC1)* contains antitaxial veins with fibrous and acicular calcite crystals (e.g., samples JB81, JB84, and JB88) (Figure 4a and Table 1). A characteristic median line by coarse, blocky quartz aggregates, and wall rock fragments is observed. *Type two (VC2)* is characterized by polymineralic filling of spherical aggregates of coarse quartz crystals in the center of the veins, surrounded by clay minerals along the vein wall and fibrous calcite (e.g., samples JB60, B71, JB74, JB78) (Figure 4b and Table 1). The radiating quartz crystals can reach a size up to 2 mm and in some cases these crystals are overgrown by small calcite grains. *Type three (VC3)* is characterized by syntaxial veins, filled with elongated crystals of aragonite with a clay selvage (e.g., samples JB59, JB62, JB63, JB66, JB69, and JB89) (Figure 4c and Table 1). Within the CCR basalt vesicles occur discretely throughout Unit 1‐6 and 8. Their abundance vary within the different units, however, highest abundance is typically within the upper portion of each unit. The common vesicle‐filling phases are saponite, smectite, pyrite, quartz, and calcite, whereby fibrous calcite constitutes the late filling phase (Figure 4d).

**Figure 5:**
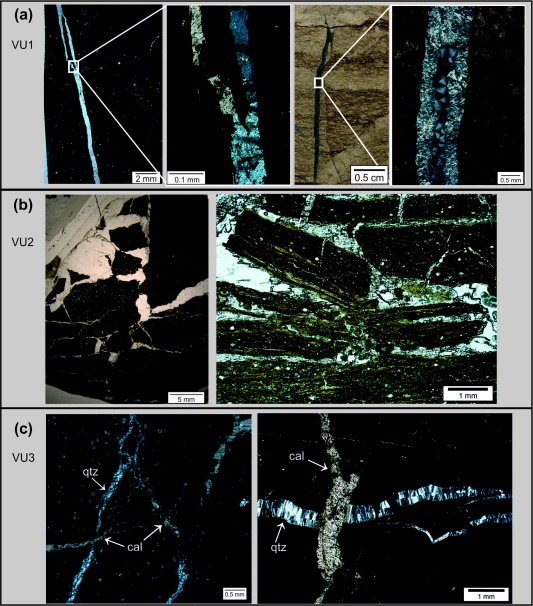
(a) Discrete veins of type VU1 filled with (left) calcite and (right) quartz. (b) Blocky vein (VU2) of sample JB58 and sample JB57 with wall rock fragments within the veins. (c) Cross‐cutting relationship between quartz veins and younger calcite veins (samples JB58 and JB43). See Table 1 for sampling codes.

In the sedimentary rocks of Unit III, the vein orientation is highly variable. Vein generations can be distinguished by cross‐cutting relationships and contrasting to veins in the CCR basalt, the veins consist mainly of calcite. Subordinately spherical aggregates of acicular quartz crystals occur. Three different vein types are distinguished. *Type one (VU1)* is characterized by discrete veins of calcite and subordinately discrete veins with different growth intervals of quartz (e.g., samples JB35, JB36, and JB37) (Figure 5a and Table 1). *Type 2 (VU2)* defines irregular, blocky veins with coarse‐grained calcite and wall rock fragments embedded within the vein filling (e.g., samples JB41, JB42, JB48, JB50, JB57, and JB58) (Figure 5b and Table 1). *Type 3 (VU3)* comprises small nondiscrete veins filled with calcite (e.g., samples JB43, JB51, and JB 58) (Table 1). In cases, type 3 crosscut type 1 veins or occur as late precipitates in the vicinity of type 2 veins (Figure 5c).

## 6 Fluid Inclusion Analyses

### 6.1 FI Characteristics in Mineralized Veins

**Figure 6:**
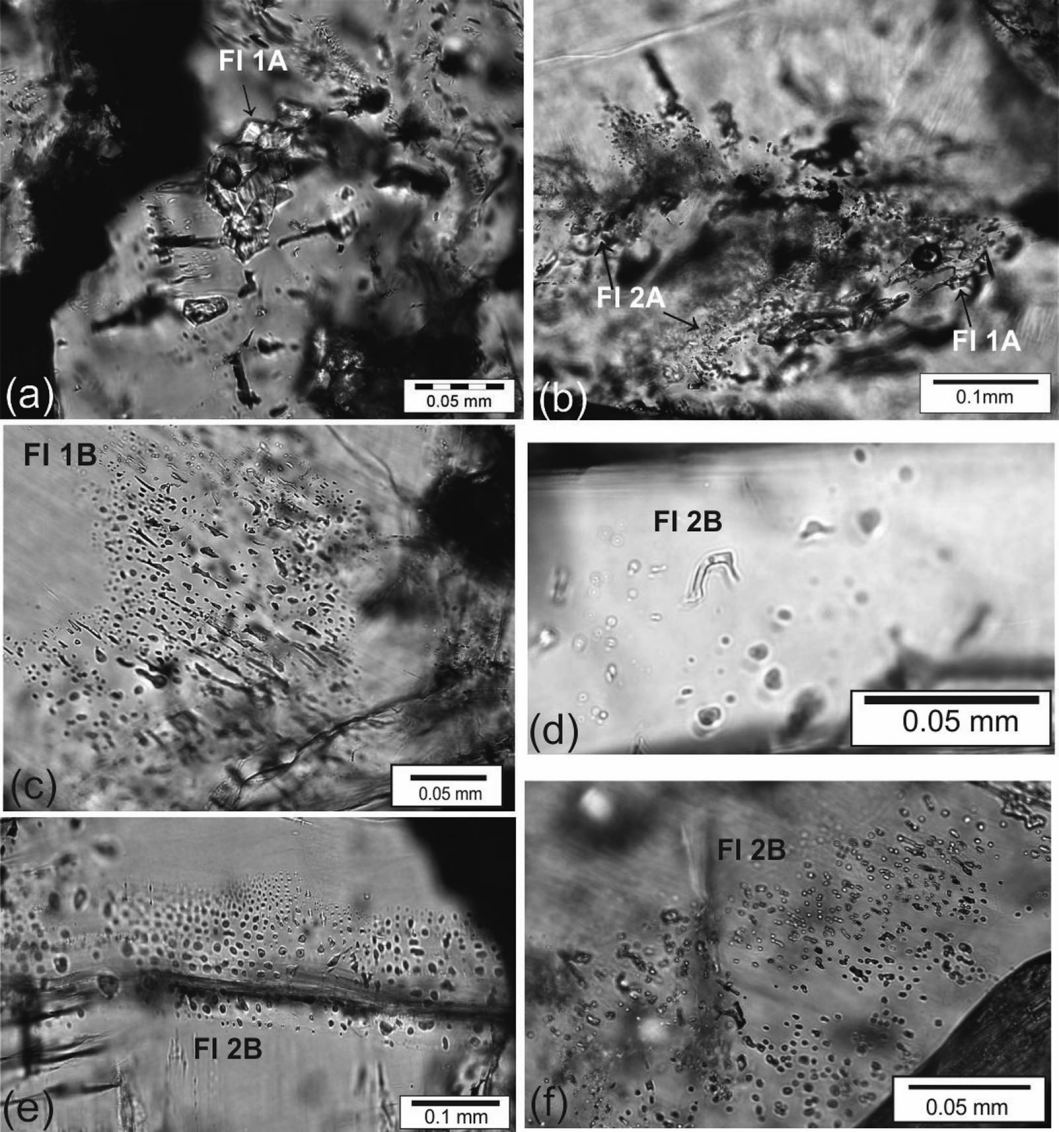
(a) Large, primary fluid inclusions of type FI 1A, with irregular shape hosting in calcite (sample JB50). (b) FI 1A with decrepitation texture surrounded by smaller FI 2A (satellites) hosting in quartz from the CCR basalt (sample JB60). (c) Elongated fluid inclusions (FI 1B) along crystal growth directions of calcite (sample JB69). (d) Arc‐like FI of FI 2B hosting in calcite (sample JB50). (e) FI 2B along crystal growth planes of aragonite (sample JB62). (f) Nearly undeformed FI 2B in quartz (vesicle of sample JB94). See Table 1 for sampling codes.

The quartz/aragonite veins (VC2/3) in the CCR basalt and the calcite veins (VU1‐3) in the lithified sediment layers of Unit III contain similar FIs. Within type VC1, no FIs were suitable for microthermometry. Two major fluid inclusion generations, primary (FI 1) and latest or secondary (FI 2), are distinguished by textural characteristics. FI 1 is further divided into subtypes FI 1A and FI 1B, based on their isolated/single appearance and FIPs with intragranular characteristics, respectively (Figures 6a–6c). FI 1A with a size from 6 to 80 µm represent an early generation with mostly irregular, sometimes arc‐like and dendritic shapes, surrounded by smaller satellite inclusions (Figure 6b). This FI type occurs rarely in quartz veins within the CCR basalt, but is the major constituent in the calcite veins of Unit III. Type FI 1B consist of FIs with a size from 6 to 25 µm, mostly elongated and arranged parallel to crystal growth directions (Figure 6c). Secondary or late FIs (FI 2) can also be distinguished into subtypes FI 2A and FI 2B, characteristic for satellite inclusions surrounding FIs from FI 1A and modified intragranular FIPs or clusters, respectively. FI 2A were entrapped due to decrepitation of FI 1A and show a size between <5 and 30 µm (Figure 6b). Their shape is mostly irregular and flat. FI 2B define FIs either showing irregular shape and arc‐like textures (Figure 6d) or late entrapped FIs with mostly undeformed and regular shapes showing an average size of <10 µm (Figures 6e and 6f).

#### 6.1.1 FI Data From Quartz Veins (VC1) of the CCR Basalt (Samples JB60 and JB94)

Due to radiating fibrous growth of the quartz host, the number and also the size of FIs are small. Two samples (vein sample JB60 and vesicle sample JB94), however, were useful for microthermometry (Table 2). In sample JB60, FIs are classified as FI 1A, 2A and 2B. Due to the low eutectic temperatures *T*_e_(Ice) of ∼−62°C a H_2_O‐NaCl fluid chemistry with additional chlorides like KCl, MgCl_2_, and CaCl_2_ is suggested. FI 1A obtains *T*_m_(Ice) of ∼−2.0°C and *T*_h_(total) between 270 and 400°C. Corresponding densities range from 0.79 to 0.49 g/cm^3^. Total salinity is about 3.4 mass%. FI 2B shows *T*_m_(Ice) of −1.5°C and a total salinity of 2.6 mass%. *T*_h_(total) ranges between 120 and 250°C, resulting into density calculations from 0.96 to 0.82 g/cm^3^, respectively. In sample JB94, FI 2B with a *T*_h_(total) ranging from 40 to 123°C yield densities between 1.0 and 0.97 g/cm^3^, respectively. *T*_m_(Ice) lies around −3.0°C, which results in a total fluid salinity of about 5.0 mass%.

#### 6.1.2 FI Data From Aragonite Veins (VC3) of the CCR Basalt (Samples JB62, JB66, JB69)

The occurrence of FI 1B is restricted to aragonite veins (VC3) in the CCR basalt. FI 1B shows a *T*_e_(Ice) from −50 to −42°C, indicative for a fluid system similar to FIs from quartz veins. *T*_m_(Ice) lies between −1.6 and 0.0°C, with salinities up to 2.7 mass%. *T*_h_(total) between 260 and 375°C results in densities ranging from 0.80 to 0.55 g/cm^3^, respectively (Table 2). FI 2B with a *T*_h_(total) ranging from 80 to 230°C yield densities between 0.98 and 0.83 g/cm^3^, respectively. *T*_m_(Ice) lies between −2.0 and 0.0°C, which results into a total fluid salinity up to 3.4 mass%.

#### 6.1.3 FI Data From Calcite Veins of Unit III (VU1‐3) (Samples JB37, JB50, JB51, JB58)

FI 1A obtain a *T*_e_(Ice) between −70 and −45°C which indicates the same fluid system as in the CCR basalt. *T*_m_(Ice) range between −2.0 and −0.5°C and *T*_h_(total) between 230 and 430°C (Table 2). Total salinities yield 0.8 to 3.4 mass% (Table 2). Calculated densities vary between 0.83 and 0.36 g/cm^3^. FI 2A obtain a *T*_h_(total) range between 230 and 370°C with corresponding densities between 0.83 and 0.55 g/cm^3^. FI 2B show a *T*_e_(Ice) between −55 and −40°C, *T*_m_(Ice) between −0.3 and −3.0°C and *T*_h_(total) between 74 and 200°C. Corresponding densities range from 0.99 to 0.89 g/cm^3^ and calculated salinities yield 0.5 to 5.0 mass%.

## 7 Discussion

At IODP Site 344‐U1414, both sedimentary and igneous rocks contain quartz‐carbonate veins with fluid inclusions of seawater or modified‐seawater composition and relatively high minimum formation temperatures. The temperature‐composition relations are very similar to those in volcanogenic massive‐sulfide deposits, which form where seawater is advected through newly formed crust or near to oceanic spreading centers (see e.g., *Zaw et al*. [2003], and the reviews by *Boschen et al*. [2013] *Hannington* [2014]). Circulation of seawater through these rocks additionally has implications for sedimentation and subsequent lithification of Unit III in terms of the tectonic setting. This is discussed in the following sections:

### 7.1 Timing of Vein Formation

Paleomagnetic data constrain the age of the CCR at Site 344‐U1381 to 14 Ma [*Barckhausen et al*., 2001]. Site 344‐U1381 is located along‐strike of the Middle America Trench about 11 km southeast from Site 344‐U1414 (Figure 1). According to *Schindlbeck et al*. [2015] Site 344‐U1381 was still in the vicinity of the CNS at that time. Biostratigraphy limits the sedimentary sequence of Unit III to the Middle Miocene, i.e., an age of ∼ 14–12 Ma [*Harris et al*., 2013a]. This therefore constrains the maximum age of vein formation. Due to the absence of lithification, mineralized veins as well as deformation structures in the units above (Units II and I, Figure 2), deformation structures within Unit III are presumed to have formed prior to the formation of Unit II, i.e., at approximately 12 Ma. The quartz/aragonite veins (VC 2/3) in the CCR basalt and the calcite veins (VU 1‐3) in Unit III show a similar fluid trend and therefore indicate a common tectonothermal evolution of these units during Middle Miocene times, close to the Galapagos hotspot and/or the CNS.

### 7.2 Fluid Inclusion Isochores and PT Path

Following the constraints described above, we calculated the litho/hydrostatic pressure conditions during vein formation and fluid entrapment within the lithified sediments of Unit III for a sedimentary cover load less than 100 m (∼66 m thickness of Unit III; absence of Units I and II) and an assumed hydrostatic load of 2000 m according to the present bathymetry. Paleopressure conditions therefore result from the sediment load (66 m × 2t/m^3^ = ∼13 bar) and the hydrostatic load (2000 m × 1t/m^3^= ∼ 200 bar), i.e., approximately 213 bar in total. This calculation includes an assumed density of 2000 kg/m^3^ for 66 m of sediments and 2000 m load of seawater with a density of 1000 kg/m^3^. By addition of 60 m of basalt with an average density of 3000 kg/m^3^ paleopressure conditions of about 233 bar are assumed for the bottom of hole U1414. For the latest fluid inclusion type FI 2B 300 m of sedimentary load (Unit I‐III) and 2500 m load of seawater were assumed, as these might have been entrapped at a position closer to the Middle America Trench, and therefore might have been already covered by Unit II and parts of Unit I. For this setting paleopressure estimates are in the range of 310 bar within Unit III and around 350 bar within the CCR basalt.

**Figure 7:**
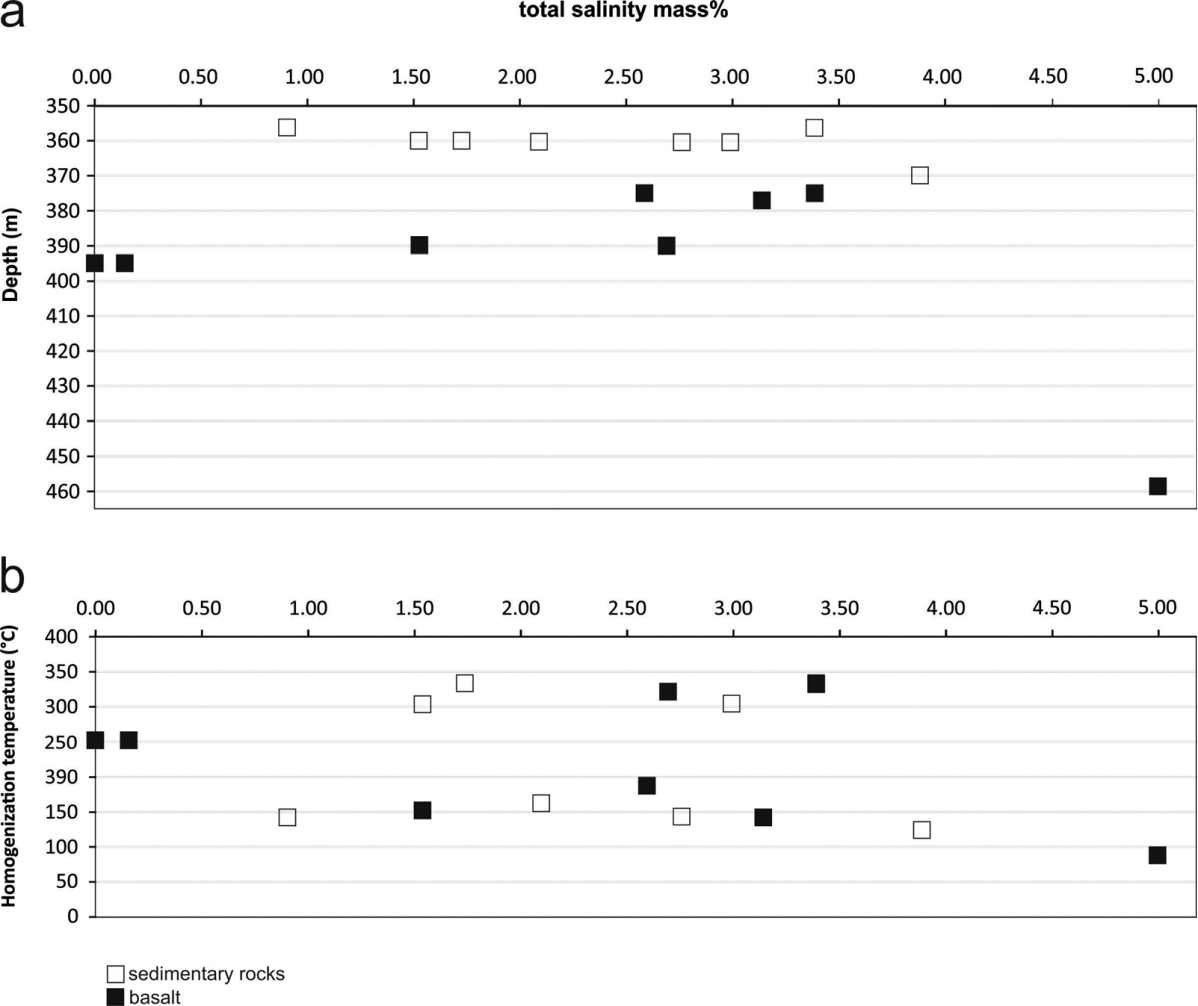
(a) Total salinity of FIs from calcite, aragonite and quartz veins of Unit III and CCR basalt. The salinity varies from 0.0 to 5.0 mass% NaCl, independently from depth (in meters below seafloor). (b) The correlation of total salinity and the homogenization temperatures of studied FIs show no clear trend. Square corresponds to the average salinity of one FI for each sample (see Table 2).

**Figure 8:**
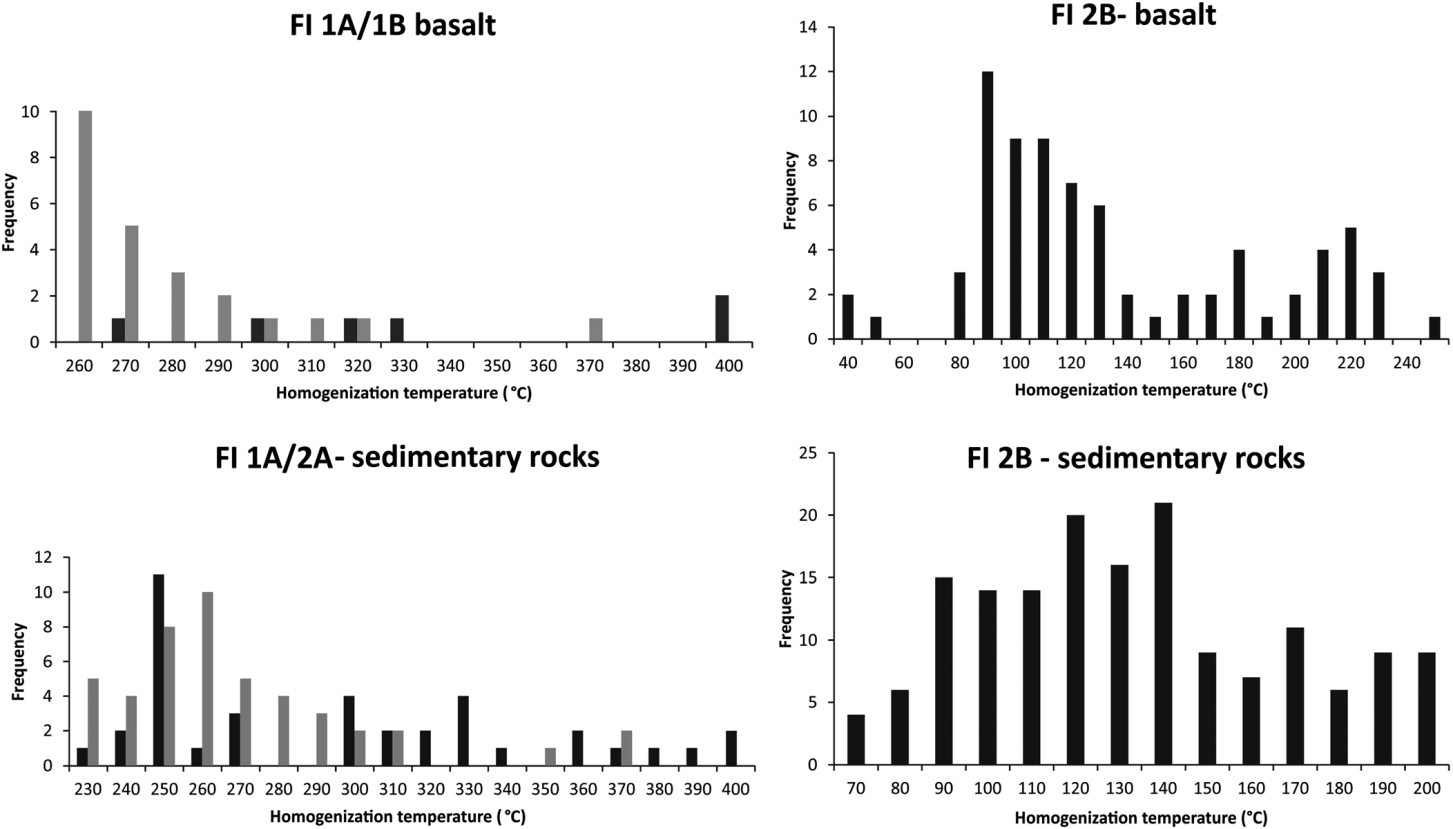
Histograms for the different fluid inclusion types in the CCR basalt and in the sedimentary rocks of Unit III. Gray bars represent homogenization temperatures of FI 1A.

**Figure 9:**
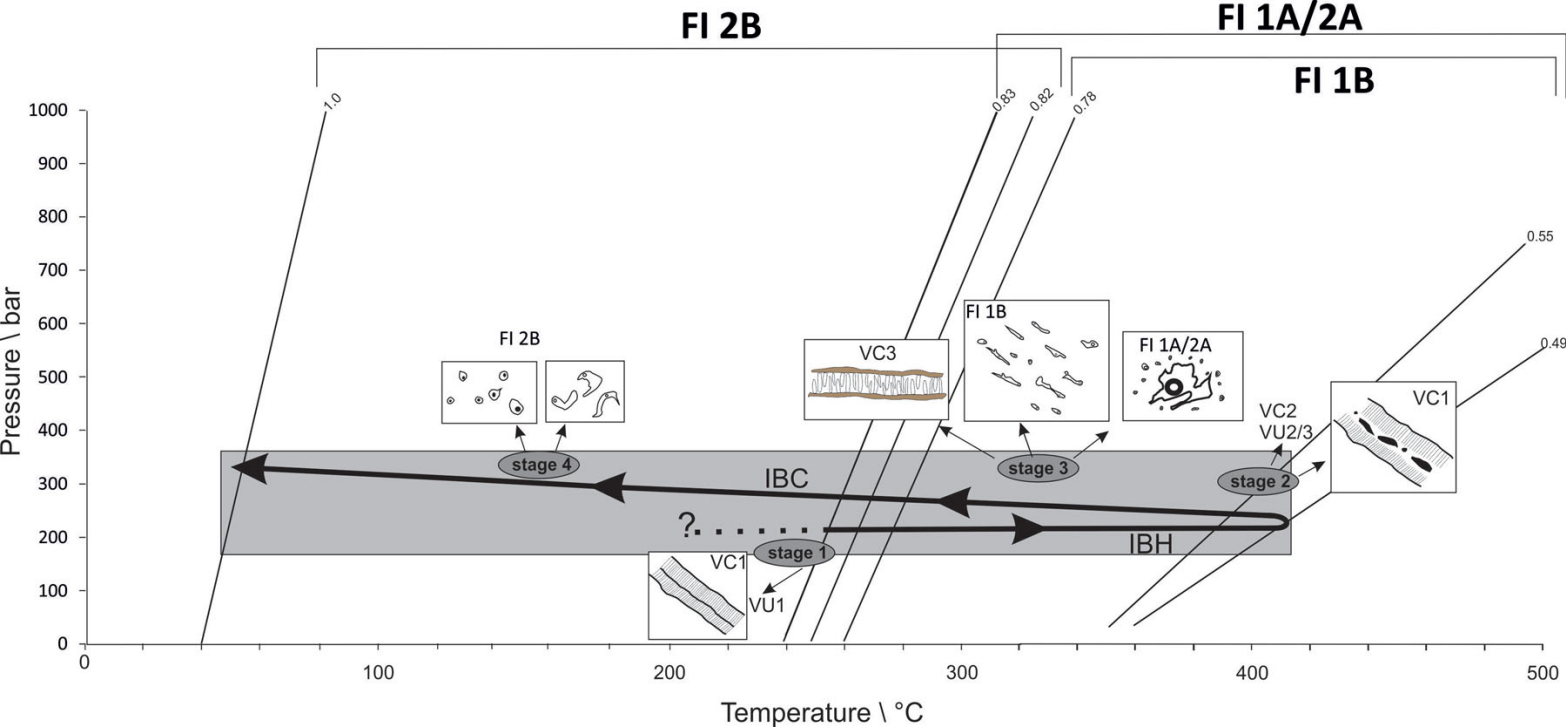
Counterclockwise P‐T path resulting from fluid inclusion isochores intersected by calculated pressure conditions (grey area—see text for detailed calculations). First vein formation of VC1 and VU1 attributed to temperatures below 240°C (stage 1, question mark). Isobaric heating path (IBH) led to decrepitation of early FIs into FI 1A and satellites (FI 2A) (stages 2/3). Internal overpressures in the FIs resulted in fractures, blocky and nondiscrete veins (VC2, VU2/3). IBH was followed by nearly isobaric cooling conditions (IBC). Stage 3 is characterized by newly formed discrete aragonite veins (VC3) with elongated FIs (FI 1B) and dendritic‐like FIs (FI 1A). Subsequent cooling modified early FIs (FI 2B) accompanied with a late fluid filling stage of vesicles (type FI 2B) (stage 4).

According to the aqueous characteristics of the FIs in all vein types, no significant change in fluid chemistry occurred during precipitation of all vein types. Additionally, no correlation between total salinity and *T*_h_(total) as well as salinity and corresponding depths of the veins is documented (Figure 7). *T*_h_(total) shows an almost similar trend with respect to the different fluid types in the CCR basalt and in the sediments of Unit III (Figure 8). Predominant steep isochores acting as thermometers were calculated (Figure 9). On the basis of almost high homogenization temperatures of FI 1A and their corresponding low densities, reequilibration of this fluid type is proposed. Surrounding satellites (FI 2A) result from modification of earlier type FI 1A. This is supported by their high range in homogenization temperatures and their density range comparable to FI 1A. Also FI 1B show lower densities, however, no evidence for decrepitation, which indicates vein formation after a stage of reequilibration. This is also valid for the arc‐like and unmodified FI 2B, which represent a late stage of vein formation, too.

Isochores crossed with estimated pressure conditions point out a PT path at almost constant litho/hydrostatic pressure conditions between 210 and 350 bar for the successive entrapment of the FIs in the veins (Figure 9). Assuming that discrete veins VC1 in the CCR basalt as well as VU1 in Unit III were formed at the earliest stage of sediment cementation, primary FI 1A should be attributed to high densities (assumed starting point stage 1 in Figure 9). This type suffered isobaric heating (IBH) that resulted in a nonisochoric PT evolution followed by subsequent reequilibration and formation of satellites (FI 2A) (stages 2/3 in Figure 9).

IBH generated internal overpressures in the early formed veins and FIs and caused hydraulic fracturing and the formation of irregular thick vein sets including quartz and clay minerals and/or wall rock fragments like type VC1/2 in the CCR basalt and VU2/3 in Unit III, respectively (Figures 4a and 5b) (stage 2 in Figure 9). FI 1B are characterized by mostly elongated, flat FIs and show also primary character. Their range in *T*_h_ displays fluid entrapment at a subsequent stage of vein formation after the temperature maximum (stage 3 in Figure 9). Subsequent to IBH, isobaric cooling (IBC) is indicated by arc‐like inclusion shape textures within VC2/3 and VU1/2 veins as result of internal underpressures in the FIs [see *Vityk and Bodnar* 1995, for comparison]. The lowest range of *T*_h_(total) from ∼120 down to 40°C is related to high density FIs in vesicles (FI 2B) which are interpreted as the latest stage of precipitation and FI entrapment (stage 4 in Figure 9). This fluid type consists of FIs with the highest salinities up to 5 mass%. The PT path characterizes an anticlockwise evolution acting in a very small pressure range. This is based on the argument that the earliest large FI 1A show a clear trend to decrepitation and their high homogenization temperatures, i.e., low densities, cannot be related to a primary origin. An isochoric PT evolution from high temperatures can be excluded by the fact that densities remain low. Considering a clockwise evolution from higher pressure conditions/deeper crustal levels followed by decompression can be excluded based on the tectonic setting (see next chapter).

### 7.3 Tectonic Model Linked With PT Evolution of the Veins

After *Barckhausen et al*. [2001] the breakup of the Farallon Plate and the subsequent opening of the CNS took place at 22.7 Ma. Major ridge jump of the CNS to the south occurred at 19.5 Ma, with shifting strike by 22°, and was followed by a second one at ∼14.5 Ma to its current orientation. The assumed cause of these ridge jumps is the interaction of the CNS with the Galapagos hotspot [*Barckhausen et al*., 2001]*. Schindlbeck et al*. [2015] interpret the strong increase in eruption frequency at ∼14 Ma as a proxy of increased plume magma production.

**Figure 10:**
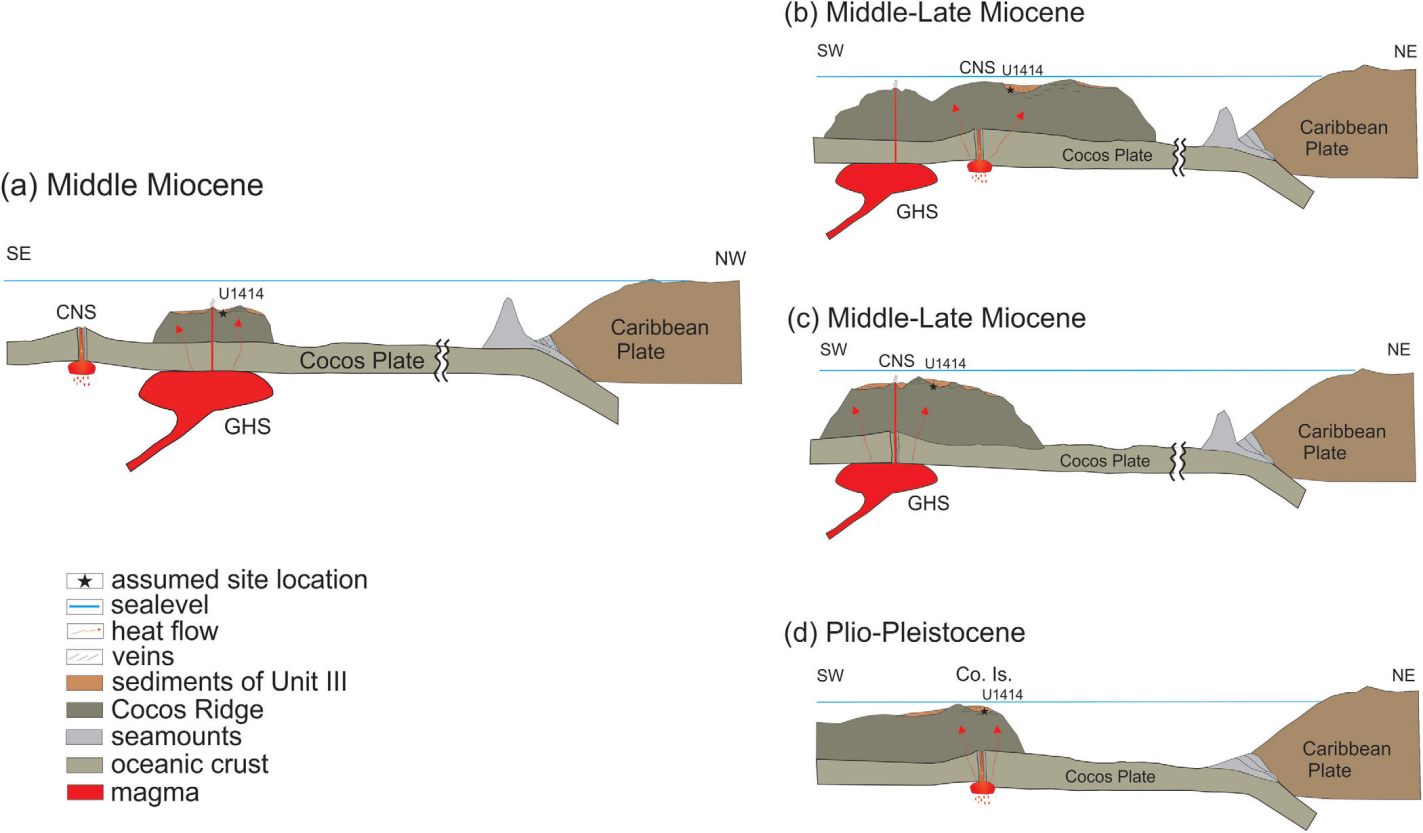
Simplified tectonic sketches of the Middle Miocene tectonic situation of the Cocos Plate (not to scale). (a) The Cocos Plate was formed at the Cocos‐Nazca spreading center (CNS) and subducted under the Caribbean Plate. The activity of the Galapagos hotspot (GHS) resulted in the formation of the Cocos Ridge. Basaltic eruptions and advective heat transfer led to lithification of sediments of Unit III accompanied with first vein formation. The Cocos Ridge and Unit III underwent heating by a second event. The heat source was either (b) the CNS, due to the ridge jump at ∼14.5 Ma, (c) a combination of CNS and GHS activity or (d) seamount volcanism in the area of Cocos Island (Co. Is.).

Vein formation, filled by clay minerals and carbonates (VC1), started within the magmatic basement of the CCR most probably in the vicinity of either the CNS or the Galapagos hotspot (Figure 10a). As the sediments of Unit III were deposited on thickened CCR crust, the latter option is preferred. No FIs were observed in this early vein sequence. However, FI 1A in quartz within VC2 indicate subsequent fluid entrapment (stage 2 in Figure 9). This early stage is related to the formation of vein type VC1 which consists of calcite bordered by clay minerals. Subsequent to vein formation in the CCR basalt fluid migration into the sedimentary sequence of Unit III led to cementation and lithification and the subsequent formation of earliest discrete calcite and quartz veins VU1. This assumption is corroborated by numerous bedding parallel layers of calcite cement within the lithified sediments (see also Figures 3a and 3c) that were transected by hydrothermal veins. Basaltic eruptions [*Schindlbeck et al*., 2015] combined with advective heat transport caused the mobilization of pore fluids within the lowermost sedimentary beds of Unit III and enabled lithification and deformation (Figure 10a).

The following stages include additional heat advection either from the Cocos‐Nazca spreading center (Figure 10b), from the interfering CNS ‐ Galapagos hotspot system (Figure 10c) or from seamount volcanism superimposed on the CCR approximately 2 Ma ago in the area of Cocos Island [*Castillo et al*., 1988; *Harpp et al*., 2005] (Figures 1 and 10d). The advection‐related isobaric heating led to internal overpressures in the lithified sediments and in the veins. The chert‐like laminae (deriving from heat‐supported silica segregation) within Unit III (Figure 3a) potentially acted as hydraulic barriers. The internal overpressures resulted in the formation of hydraulic fractures (VC2, VU2) and into reequilibration of early FIs (FI 1A).

The formation of an irregular fracture network due to IBH enabled the infiltration of seawater to mingle it with mobilized pore fluids and hydrothermal fluids. This may be indicated by total salinities of the FIs between 0 and ∼5 mass% (average salinity of sea water is about 3.5%). Mingling is supported by ^87^Sr/^86^Sr ratios from pore fluids sampled from sequences of Unit III [*Ross et al*., 2015], which show evidence of ash alteration and increasing carbonate diagenesis and cementation with depth. The deepest sediments here, however, may also have been modified by diffusion of hydrothermal fluids from the underlying CCR basalt [*Ross et al*., 2015]. Moreover, Ca and Li isotope analysis of sediment pore water indicates communication with high‐temperature fluids in the range of ∼300–350°C and the involvement of a hydrothermal system [*Harris et al.,* 2012] [see *Harris et al*., 2013b for data].

Under almost isobaric cooling conditions (IBC), small discrete aragonite veins (VC3) were formed (stage 3 in Figure 9) and late calcite precipitation in vesicles in the CCR basalt goes along together with modification of FIs within the veins (stage 4 in Figure 9). These stages probably last up to recent times with hole U1414 situated close to the Middle America Trench.

## 8 Conclusions

1. At the time of initial vein formation, the present Site 344‐U1414 was located close to the Galapagos hotspot and/or the Cocos‐Nazca spreading center. A maximum Middle Miocene time constraint for initial vein formation is given by the sedimentation age of Unit III and the base of Unit II.
2. Advective heat transport, probably related to the Galapagos hotspot, effectuated lithification of Unit III.
3. The source of fluids within hydrothermal veins is a mixture of mobilized pore water and invaded seawater. Fluid inclusion analyses and referenced isotope data show evidence of communication with deeper sourced, high‐temperature hydrothermal fluids within the Cocos Plate magmatic basement.
4. The fluid inclusions document a multistage evolution from fluid entrapment, isobaric heating, a stage of internal fluid overpressure and subsequent isobaric cooling.
5. Veins that reveal multiple episodes of fluid entrapment and modification suggest that fluid flow occurred over several episodes, possibly up to recent times.
6. Fluids that were generated during Middle Miocene time were recently and will be incorporated into the subduction system of the CRISP study area. Hence, they will become part of the hydraulic system within the subduction channel and may be released into the fracture system of the upper plate.
